# Gynaecologic cancer care during COVID‐19 pandemic in India: a social media survey

**DOI:** 10.1002/cnr2.1280

**Published:** 2020-09-17

**Authors:** Sarita Kumari

**Affiliations:** ^1^ Department of Obstetrics and Gynaecology All India Institute of Medical Sciences New Delhi India

**Keywords:** COVID‐19 pandemic, developing country, health care, survey

## Abstract

**Background:**

Health care services across the globe are undergoing a major transformation to combat the coronavirus disease 2019 (COVID‐19) pandemic. Regardless of the strength of health care infrastructure across different economies, all countries are diverting their resources toward care for COVID‐19 patients.

**Aim:**

The aim of this survey was to evaluate the pattern of care of gynaecologic cancers in a developing country during the COVID‐19 pandemic.

**Methods:**

An anonymous survey consisting of 20 questions intended for the gynaecologic cancer care providers with emphasis on their current practice and approach to their patients was distributed online via social media from April 30 to May 31, 2020. Basic descriptive statistics were applied.

**Results:**

Among a total of 61 respondents, 63.9% were gynaecologic oncologists, 18.0% were radiation oncologists and 18.0% were medical oncologists. Majority, that is, 95.1% health care professionals felt that COVID‐19 pandemic has had a significant change on their practice pattern and 56.2% practitioners had stopped registering new cases of cancer. In 75.4% centers surgery was being done for gynaecologic cancer cases and among them 60.8% were doing surgery only for cases requiring immediate intervention. Among the centers providing chemotherapy, 39.1% had switched to oral drugs. Among the centers providing radiation, 40.9% were providing radiation to cases based on their type and urgency and 9.0% had implemented hypofractionation. In early stage low risk cases, majority, that is, 34.0% centers were managing as before. In early stage high‐risk cases, 32.6% centers were managing as before. In advanced stage endometrial cancer cases, 28.8% had postponed any treatment and 28.8% administered chemotherapy. In early stage, epithelial ovarian cancer 65.9% centers were performing complete staging of the disease. In advanced stage epithelial ovarian cancer, 65.9% centers preferred biopsy followed by neoadjuvant chemotherapy and 11.3% centers performed primary debulking surgery. In cases of interval debulking surgery, 73.3% centers deferred surgery till all six cycles of chemotherapy was completed. In cases of recurrent ovarian cancer amenable for secondary debulking surgery, 38.6% preferred chemotherapy. In early stage cervical cancer, surgical treatment was provided in 46.5% centers. In locally advanced cervical cancer, chemoradiation was given in 65.9% centers. In cases of metastatic cervical cancer, 46.6% centers were performing palliative radiation.

**Conclusion:**

COVID‐19 has affected the treatment of gynecologic cancers patients and health care professionals are trying to mitigate the damage by incorporating new elements which are suited to the current scenario.

## 1 INTRODUCTION

Coronavirus disease 2019 or COVID‐19 caused by severe acute respiratory syndrome coronavirus 2 (SARS‐CoV‐2) has emerged as a pandemic affecting more than 200 countries and territories around the globe. It has impacted all areas of daily life including medical care. The WHO has defined priority areas for all countries which include protecting health workers, engaging communities to protect those at highest risk of severe disease and supporting vulnerable countries in containing infection.^1^ Across the world, health care systems are coming up with new proposals and guidelines to tackle the disease.^2^ In a developing economy such as India, lack of adequate health infrastructure (limited hospital capacity and ICUs, inadequate supplies of personal protective equipment) compounded by fear among patients and health care workers has severely compromised the patient care.

Cancer care has suffered this set back particularly as majority of health care has been diverted to COVID‐19 care and cancer centers have scaled back their services after studies from China showing significantly worse outcomes of COVID‐19 among cancer patients. Patients with cancer are struggling to receive treatment. Even in patients having curable cancer and without any comorbidities, who will derive therapeutic benefit form timely care, treatment is being postponed considering the risk of contracting COVID‐19 may outweigh the benefits of cancer treatment. Several medical societies have come up with guidelines for oncology care providers during pandemic.^3^, ^4^, ^5^ However, resources addressing the quality of current practice remain scarce. Amidst all these dilemmas we thought of implementing a survey to see the pattern of cancer care among gynaecologic cancer cases in India.

## 2 METHODS

We constructed a survey consisting of 20 questions intended for the gynaecologic cancer care providers with emphasis on their current practice and approach to their patients. Freely available online survey tool (www.surveymonkey.com) was used to create the survey. The survey was structured to capture general anonymous data of respondents. It was up to the respondents to choose whether or not to answer any question in the survey, thus each question was not necessarily answered by all respondents. The percentages were counted from those who answered a certain question and not from the entire cohort (the denominator was the number of respondents to each single question). Moreover, some questions allowed multiple options to be selected. Questions were directed to the most common gynaecologic cancers, that is, carcinoma endometrium, carcinoma ovary and carcinoma cervix and the results are presented here. On April 30, after creating a social media link the survey was sent to gynaecologic oncologists, medical oncologists and radiation oncologists across all hospitals catering to cancer patients in different states through email, Twitter, Facebook and Whatsapp and the survey was closed on May 31, 2020.

### 2.1 Statistical analysis

Data were analyzed using inbuilt software in the survey tool. Descriptive statistics in terms of frequency and percentage were used to analyze the results of this study.

## 3 RESULTS

### 3.1 Characteristics of respondents

There were a total of 61 respondents. All of them were health professionals involved in managing gynaecologic cancer cases in different centers across the country. Majority of them, 39 (63.9%) were surgeons and remaining 11 (18.0%) were radiation oncologists and 11 (18.0%) were medical oncologists. Fourty five (73.7%) were working in government hospitals and 16 (26.2%) in private hospitals. At the time of survey 20 (32.7%) respondents said that their hospital was free from COVID‐19 cases. Twenty three (37.7%) hospitals were admitting patients of COVID‐19 and rest 18 (29.5%) hospitals were following a different pathways of care for COVID free and COVID‐19 patients. Ten cancer providers had managed COVID‐19 case at the time of survey.

### 3.2 Major changes in gynaecologic cancer care

Fifty eight (95.1%) health care professionals felt that COVID‐19 pandemic has had a significant change on their practice pattern whereas three (4.9%) respondent faced no changes in their current practice. Twenty seven (56.2%) practitioners had stopped registering new cases of cancer while 21 (43.7%) were still registering new cases.

### 3.3 Changes in surgery, chemotherapy, and radiation protocols

**FIGURE 1:**
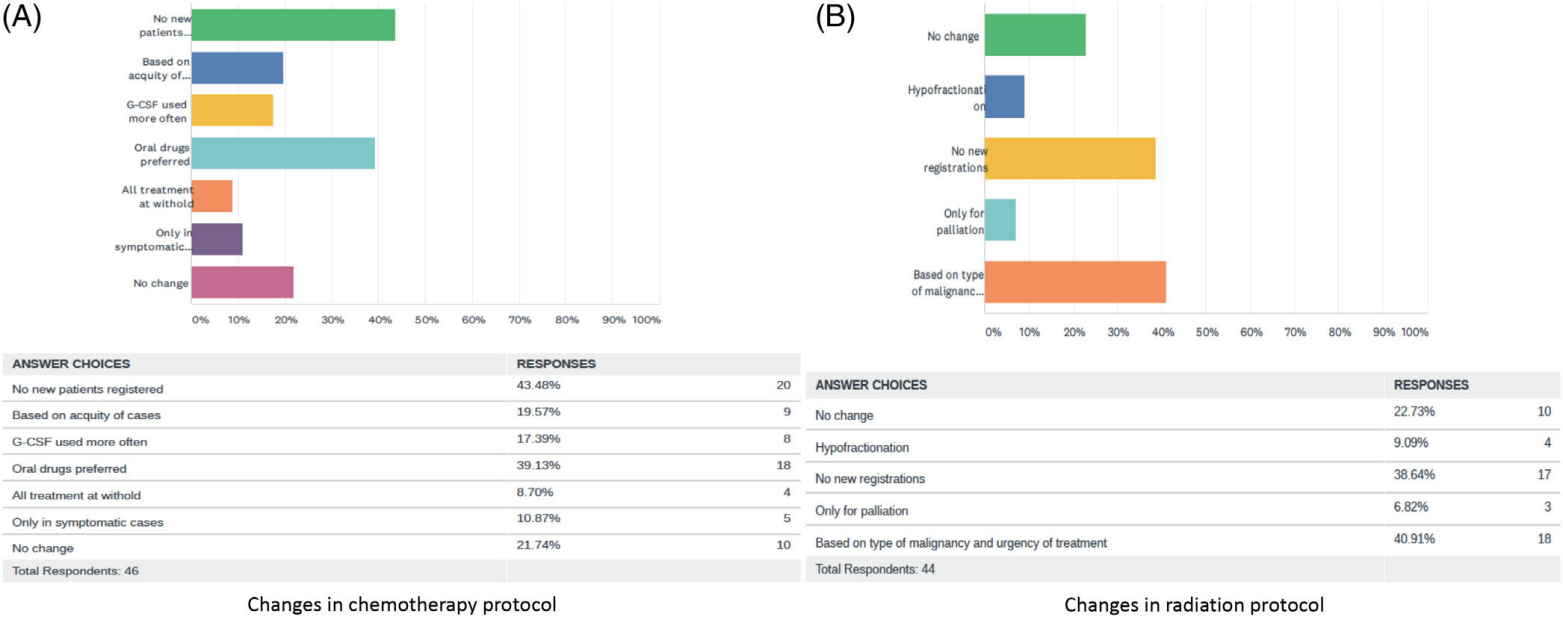
Changes in chemotherapy and radiation protocol for all gynaecologic cancer types

In 46 (75.4%) centers, surgery was being done for gynaecologic cancer cases. Among them 28 (60.8%) hospitals were doing surgery only for cases requiring immediate intervention and rest 18 (39.2%) centers were triaging cases based on the type of malignancy and urgency of surgical management. Several changes in chemotherapy protocol were made in different hospitals. Among the 46 centers providing chemotherapy, 20 (43.8%) hospitals were not registering any new patient for chemotherapy, 9 (19.5%) were administering chemotherapy based on acquity of cases, 8 (17.3%) were using granulocyte colony stimulating factors (G‐CSF) more often, 18 (39.1%) had switched to oral drugs to minimize hospital visits, 4 (8.7%) had kept all treatment on hold, 5 (10.8%) were giving chemotherapy only in symptomatic cases and 10 (21.7%) centers did not implement any changes in their protocol. Among the 44 centers providing radiation, 18 (40.9%) were providing radiation to cases based on their type and urgency, 17 (38.6%) were not registering new cases, 10 (22.3%) were functioning as before, four (9.0%) had implemented hypofractionation to reduce the number of hospital visits and three (6.8%) were only providing palliative radiation. Changes in chemotherapy and radiation protocols are depicted in Figure 1

### 3.4 Changes in treatment protocol among different cancer types:

#### 3.4.1 Carcinoma endometrium

**FIGURE 2:**
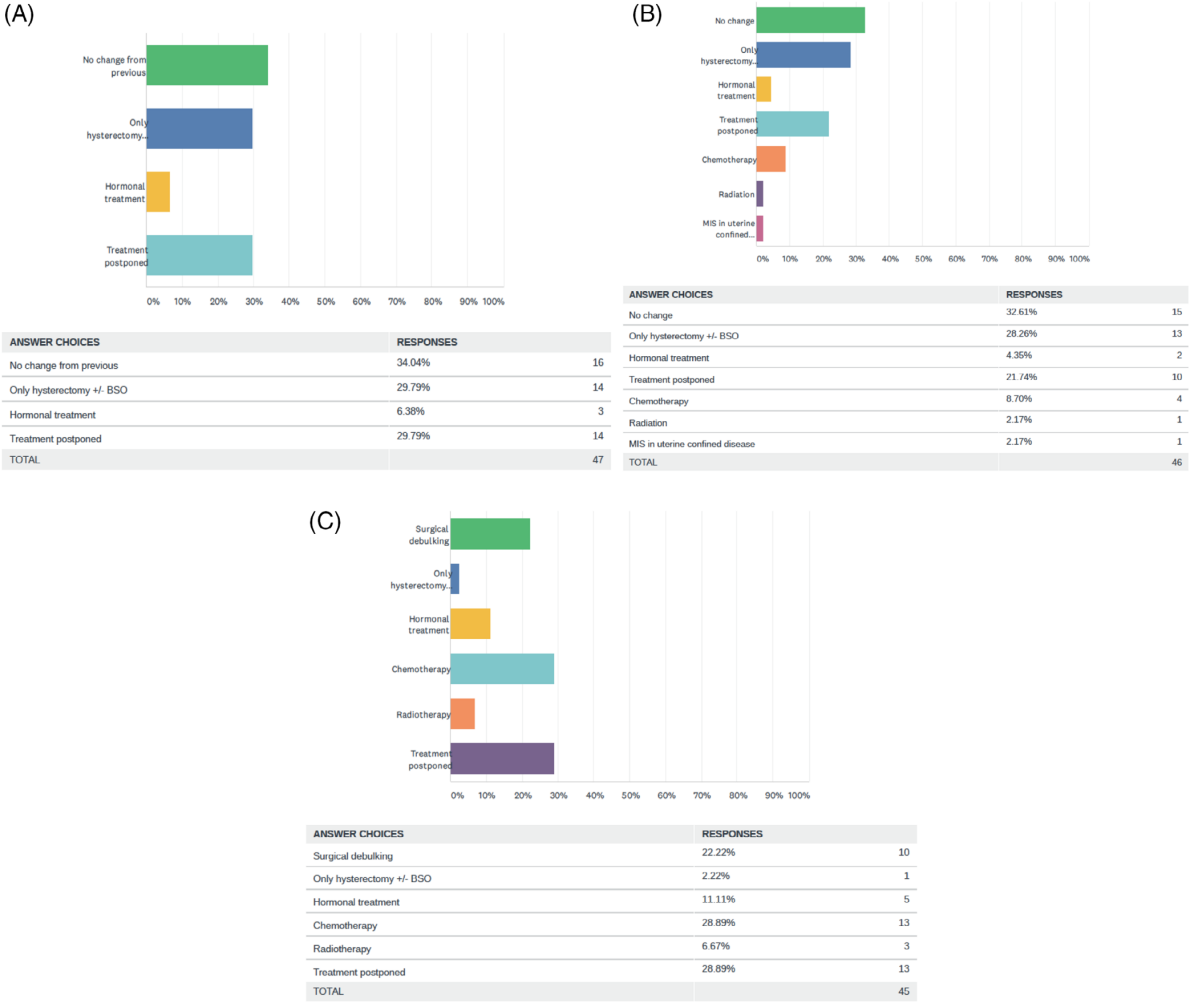
Pattern of care in endometrial carcinoma, A, early stage low risk, B, early stage high risk, and C, advanced stage

In early stage low risk cases, majority, that is, 16 (34.0%) centers were managing as before, 14 (29.7%) were doing only hysterectomy with or without salpingo‐oophorectomy, 14 (29.7%) postponed treatment and three (6.3%) centers switched to hormonal treatment. In early stage high risk cases, 15 (32.6%) centers were managing as before, 13 (28.2%) were only performing hysterectomy with or without salpingo‐oophorectomy, 10 (21.7%) postponed the treatment, 4 (8.7%) were giving chemotherapy, 2 (4.3%) switched to hormonal treatment, 1 (2.1%) center was doing minimally invasive surgery in uterine confined disease and 1 (2.1%) center was administering radiation as treatment modality. In advanced stage endometrial cancer cases, 10 (22.2%) were performing surgical debulking, 13 (28.8%) had postponed any treatment, 13 (28.8%) administered chemotherapy, 5 (11.1%) gave hormonal treatment, 3 (6.6%) gave radiation and 1 center (2.2%) performed only hysterectomy with or without salpingo‐oophorectomy. Figure 2 depicts the above findings.

#### 3.4.2 Carcinoma endometrium

In early stage low risk cases, majority, that is, 16 (34.0%) centers were managing as before, 14 (29.7%) were doing only hysterectomy with or without salpingo‐oophorectomy, 14 (29.7%) postponed treatment and three (6.3%) centers switched to hormonal treatment. In early stage high risk cases, 15 (32.6%) centers were managing as before, 13 (28.2%) were only performing hysterectomy with or without salpingo‐oophorectomy, 10 (21.7%) postponed the treatment, 4 (8.7%) were giving chemotherapy, 2 (4.3%) switched to hormonal treatment, 1 (2.1%) center was doing minimally invasive surgery in uterine confined disease and 1 (2.1%) center was administering radiation as treatment modality. In advanced stage endometrial cancer cases, 10 (22.2%) were performing surgical debulking, 13 (28.8%) had postponed any treatment, 13 (28.8%) administered chemotherapy, 5 (11.1%) gave hormonal treatment, 3 (6.6%) gave radiation and 1 center (2.2%) performed only hysterectomy with or without salpingo‐oophorectomy. Figure 2 depicts the above findings.

**FIGURE 3:**
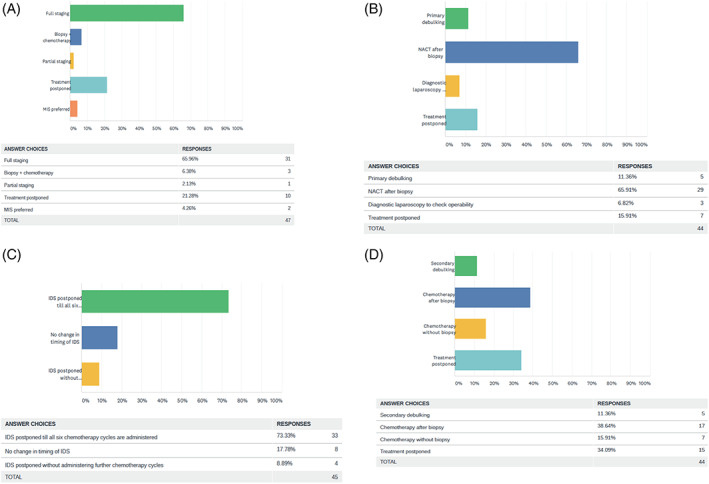
Pattern of care in carcinoma ovary, A, early stage, B, advanced stage, C, cases for interval debulking surgery (IDS), D, recurrent cases amenable for secondary debulking

#### 3.4.3 Carcinoma cervix

**FIGURE 4:**
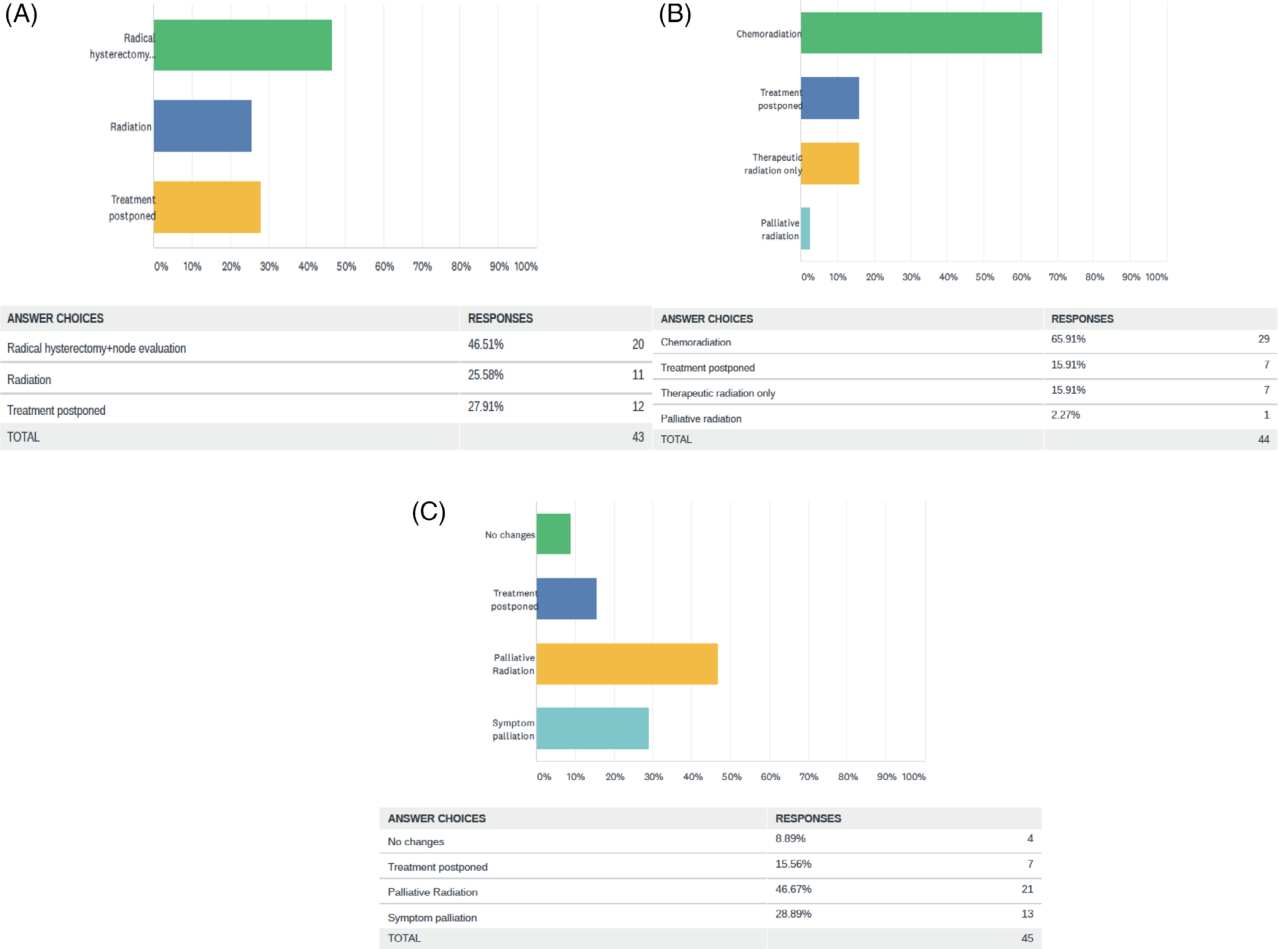
Pattern of care in carcinoma cervix, A, early stage, B, locally advanced and, C, metastatic cases

In early stage cervical cancer, treatment provided was radical hysterectomy and node evaluation in 20 (46.5%) centers, 12 (27.9%) postponed treatment and 11 (25.5%) performed radiation as primary treatment modality. In locally advanced cervical cancer, chemoradiation was given in 29 (65.9%) centers, 7 (15.9%) centers postponed treatment, 7 (15.9%) gave only therapeutic radiation and 1 (2.2%) center gave only palliative radiation. In cases of metastatic cervical cancer, 21 (46.6%) centers were performing palliative radiation, 13 (28.8%) only did symptom palliation, 7 (15.5%) postponed the treatment and 4 (8.8%) made no changes in the treatment protocol. Figure 4 depicts the above findings.

### 3.5 Changes in follow up care of patients

Fourty‐eight centers were doing follow up of their patients. Among them 20 (41.6%) switched to telemedicine, 13 (27.8%) were doing triage by phone call, 10 (20.8%) were only catering to symptomatic or suspected relapse cases and remaining 5 (10.4%) were following up as before.

## 4 DISCUSSION

Results of this survey depicts that COVID‐19 has modified the pattern of care of patients with gynaecologic cancer. A total of 95.1% respondents felt that COVID‐19 pandemic has had a significant change on their practice pattern. Nonetheless, management of COVID‐19 was quite heterogeneous. Of note, 56.2% practitioners had stopped registering new cases of cancer altogether raising questions regarding the preparedness in facing such a pandemic but other factors like availability/shortage of resources might have contributed. According to published data from Chinese hospitals, patients with cancer have a higher risk of COVID‐19 and outcomes are less favorable.^6^, ^7^ This might have been the major reason of postponing the treatment. Several authors have recommended delaying surgical procedures as surgery might increase the risk of developing covid‐19 infection.^8^ Bogani et al conducted a retrospective study including all consecutive patients affected by gynecological cancer who developed COVID‐19 in an academic setting in Milan, Italy between February and March 2020. Of 355 patients with gynaecologic cancer, newly diagnosed or recurrent, 19 (5.3%) developed COVID‐19. All patients were asymptomatic at the time of COVID‐19 detection. Six patients were diagnosed before starting planned treatments; while the remaining 13 were diagnosed for COVID‐19 after they started their treatment. In the first group of six patients, one patient died due to COVID‐19, 3 days after the diagnosis. The latter group of 13 patients (treatments started) included five patients who underwent surgery and eight patients who underwent chemotherapy. Of the five patients who were diagnosed after surgery, they observed that two patients died during postoperative course, while in other two cases prolonged hospitalization was needed. They concluded that COVID‐19 impacts the quality of treatments for cancer patients.^9^ It is well established that abdominal surgical procedures (especially if they are performed via open approach) per se, are associated with a high risk of postoperative pulmonary complications.^10^ Bogani et al in their review on gynaecologic oncology in times of COVID‐19 suggested that extensive surgical procedures for which the admission of intensive care unit would be necessary should be taken in consideration only in selected cases, while unfit and elderly patients should receive the possible less invasive procedures. A thorough consideration of patients' performance score, disease characteristics as well as prognosis should be taken into account.^11^

Fortunately, 32.7% and 29.5% respondents worked in COVID‐19 free hospitals or in hospitals where different pathways of care for COVID free and COVID‐19 patients was being followed. Only 10 cancer providers had managed COVID‐19 case at the time of survey depicting the early phase of pandemic in the country. Globally, surgical practice (60.8% hospitals doing surgery only for cases requiring immediate intervention), medical oncology (43.8% hospitals not registering any new patient for chemotherapy, 39.1% had switched to oral drugs) and radiation oncology (40.9% providing radiation to cases based on their type and urgency, and 38.6% not registering new cases) were impacted by COVID‐19.

Being a curable cancer, early stage low risk cases endometrial cancer was being managed as before in majority, that is, 34.0% centers. Similar approach was seen in early stage high‐risk cases, where 32.6% centers were managing as before. In contrast to above, in advanced stage endometrial cancer, majority were given chemotherapy (28.8% each). Early stage epithelial ovarian cancer was also treated with and intent of giving the maximum survival benefit to the patients (65.9% centers were performing complete staging of the disease). In advanced stage epithelial ovarian cancer, 65.9% centers preferred biopsy followed by neoadjuvant chemotherapy and only 11.3% centers performed primary debulking surgery. Aim was mainly to limit the surgical morbidity and decrease the time of stay in hospital. With a similar intent, 73.3% centers deferred surgery till all six cycles of chemotherapy was completed and secondary debulking surgery was being performed only in 11.3% centers despite its survival benefit. In a review on management of patients ovarian cancer in COVID era, authors suggested that in the early stage disease, surgery should be considered mandatory. In patients with advanced stage disease at diagnosis, the use of primary cytoreductive surgery should be carefully considered. When extensive surgical procedures are anticipated in this setting, surgery should probably be avoided.^12^ In early stage cervical cancer, only 27.9% had postponed treatment and 11 (25.5%) performed radiation as primary treatment modality to avoid surgical morbidity. In locally advanced cervical cancer, chemoradiation was given in 65.9% centers considering the risk benefit ratio of treating or not treating the disease. In Metastatic cervical cancer, only palliative treatment was considered in view of poor prognosis. Follow up of patients was switched to teleconsultation in majority (41.6% centers). Indini et al in their nationwide survey on reorganization of medical oncology departments in Italy highlighted the importance of treatment tailoring. They advocated the use of teleconsultation services and reducing the number of visits by means of customizing treatment delivery.^13^

In the period of lock‐down, social media became a reliable tool to stay in contact with colleagues from around the world. With this survey, we tried to evaluate through social media the impact of the COVID‐19 pandemic on gynecologic cancer care in a developing country. There are limitations of this survey. As COVID‐19 pandemic is a rapidly evolving situation and practices are changing rapidly, response registered at the time of survey might change afterwards. Across all centers, cancer‐specific treatments are not standardized (eg, use of neoadjuvant chemotherapy vs primary debulking surgery for advanced ovarian cancer, type of nodal staging in uterine cancers, indication for primary surgery vs chemoradiation therapy for cervical cancer). This could have affected the responses but it also depicts the real world practice.

## 5 CONCLUSION

COVID‐19 pandemic has affected the way professionals manage patients with cancer. Moreover, the common feeling of uncertainty prevails. Rapid and difficult decisions are being made in response to the pandemic which is straining already fragile health care system. Elements of cancer care are being redefined taking into account the risk benefit ratio to patients as well as health care professionals. Nevertheless, as we can see from this survey that health care professionals are trying to mitigate the damage by incorporating new elements which are suited to the current scenario.

## AUTHOR CONTRIBUTIONS

**Sarita Kumari:** Conceptualization; investigation; methodology; resources; software; supervision; validation; visualization.

## CONFLICT OF INTEREST

The authors have stated explicitly that there are no conflicts of interest in connection with this article.

## ETHICS STATEMENT

Completion of survey was considered as written informed consent for participation.

6

## Data Availability

The data that support the findings of this study are available from the corresponding author upon reasonable request.

## References

[1] World Health Organization . Director‐General's opening remarks at the media briefing on COVID‐19, February 24, 2020 https://www.who.int/dg/speeches/detail/who‐director‐general‐s‐opening‐remarks‐at‐the‐media‐briefing‐on‐covid‐19‐24‐february‐2020 (accessed on February 26, 2020).

[2] https://www.cdc.gov/coronavirus/2019‐ncov/healthcare‐facilities/guidance‐hcf.html (accessed on April 2, 2020).

[3] COVID‐19 Patient Care Information, American Society of Clinical Oncology (ASCO) . https://www.asco.org/asco-coronavirus-information/care-individuals-cancer-during-covid-19 (accessed on April 2, 2020).

[4] https://www.nccn.org/covid-19/default.aspx (accessed on April 16, 2020).

[5] https://www.esmo.org/guidelines/cancer-patient-management-during-the-covid-19-pandemic (accessed on April 16, 2020).

[6] Liang W , Guan W , Chen R , et al. Cancer patients in SARS‐CoV‐2 infection: a nationwide analysis in China. Lancet Oncol. 2020;21(3):335‐337. 32066541 10.1016/S1470-2045(20)30096-6 PMC7159000

[7] Yu J , Ouyang W , Chua MLK , Xie C . (2020) SARS‐CoV‐2 transmission in patients with cancer at a tertiary care hospital in Wuhan. China JAMA Oncol. 2020;25:e200980. 10.1001/jamaoncol.2020.0980 PMC7097836 32211820

[8] Bogani G , Raspagliesi F . Minimally invasive surgery at the time of COVID‐19: the OR staff needs protection. J Minim Invasive Gynecol. 2020;27(5):1221. 10.1016/j.jmig.2020.04.010 PMC7154518 32294548

[9] Bogani G , Ditto A , Bosio S , Brusadelli C , Raspagliesi F . Cancer patients affected by COVID‐19: experience from Milan, Lombardy. Gynecol Oncol. 2020; S0090‐8258(20)31976‐4. 10.1016/j.ygyno.2020.06.161 PMC7286266 32534808

[10] Bogani G , Rossetti D , Ditto A , et al. Minimally invasive surgery improves short‐term outcomes of nerve‐sparing radical hysterectomy in patients with cervical cancer: a propensity‐matched analysis with open abdominal surgery. J Gynecol Oncol. 2019;30:e27. 30740958 10.3802/jgo.2019.30.e27 PMC6393638

[11] Bogani G , Brusadelli C , Guerrisi R , et al. Gynecologic oncology at the time of COVID‐19 outbreak. J Gynecol Oncol. 2020;31(4):e72. 32458597 10.3802/jgo.2020.31.e72 PMC7286755

[12] Bogani G , Casarin J , Pinelli C , et al. Management of patients with ovarian cancer in the COVID‐19 era. J Surg Oncol. 2020;122:122‐123. 10.1002/jso.26057. 32476155 PMC7300981

[13] Indini A , Aschele C , Cavanna L , et al. Reorganisation of medical oncology departments during the novel coronavirus disease‐19 pandemic: a nationwide Italian survey. Eur J Cancer. 2020;132:17‐23.3231164310.1016/j.ejca.2020.03.024

